# Systematic Comparison of Constitutive Promoters and the Doxycycline-Inducible Promoter

**DOI:** 10.1371/journal.pone.0010611

**Published:** 2010-05-12

**Authors:** Jane Yuxia Qin, Li Zhang, Kayla L. Clift, Imge Hulur, Andy Peng Xiang, Bing-Zhong Ren, Bruce T. Lahn

**Affiliations:** 1 School of Life Sciences, Northeast Normal University, Changchun, China; 2 Howard Hughes Medical Institute, Department of Human Genetics, University of Chicago, Chicago, Illinois, United States of America; 3 Center for Stem Cell Biology and Tissue Engineering, Sun Yat-sen University, The Key Laboratory for Stem Cells and Tissue Engineering, Ministry of Education, Guangzhou, China; New Mexico State University, United States of America

## Abstract

Constitutive promoters are used routinely to drive ectopic gene expression. Here, we carried out a systematic comparison of eight commonly used constitutive promoters (SV40, CMV, UBC, EF1A, PGK and CAGG for mammalian systems, and COPIA and ACT5C for *Drosophila* systems). We also included in the comparison the TRE promoter, which can be activated by the rtTA transcriptional activator in a doxycycline-inducible manner. To make our findings representative, we conducted the comparison in a variety of cell types derived from several species. We found that these promoters vary considerably from one another in their strength. Most promoters have fairly consistent strengths across different cell types, but the CMV promoter can vary considerably from cell type to cell type. At maximal induction, the TRE promoter is comparable to a strong constitutive promoter. These results should facilitate more rational choices of promoters in ectopic gene expression studies.

## Introduction

Many constitutive promoters are used to drive ectopic gene expression in various *in vitro* and *in vivo* contexts. While a number of studies have evaluated the strengths of these promoters in various cellular contexts [1], [2], [3], [4], [5], [6], [7], there is generally a shortage of systematic comparisons of the behaviors of commonly used constitutive promoters across multiple cell types under the same experimental conditions. The choice of which promoter to use is therefore frequently based on technical convenience, such as the availability of a promoter, rather than the suitability of the promoter for a particular experiment. To facilitate more rational choices of promoters in ectopic gene expression studies, we decided to examine six constitutive promoters commonly used in mammalian systems, including the simian virus 40 early promoter (SV40), cytomegalovirus immediate-early promoter (CMV), human Ubiquitin C promoter (UBC), human elongation factor 1α promoter (EF1A), mouse phosphoglycerate kinase 1 promoter (PGK), and chicken β-Actin promoter coupled with CMV early enhancer (CAGG). We also chose to examine two promoters commonly used in *Drosophila* systems, including copia transposon promoter (COPIA) and actin 5C promoter (ACT5C). Finally, we included in the comparison the doxycycline-inducible system with reverse tetracycline-controlled transactivator (rtTA) and tetracycline-responsive element promoter (TRE).

We investigated these promoters in multiple cell lines derived from several species to get a general sense of their behaviors. For the mammalian promoters, we used mouse tail fibroblasts (129TF), mouse embryonic fibroblasts (MEF), mouse myoblasts (C2C12), rat mesenchymal stem cells (MSC), human fibroblasts (MRC5), human fibrosarcoma cells (HT1080), human embryonic kidney cells (293T), and rhesus macaque mammary tumor cells (CMMT). For *Drosophila* promoters, we used two *Drosophila melanogaster* cell lines, including the ML-DmBG3-c2 cell line (C2) derived from larval central nervous system [8] and the S2R+ cell line (S2R+) derived from late embryo [9], [10].

## Results and Discussion

**Figure 1:**
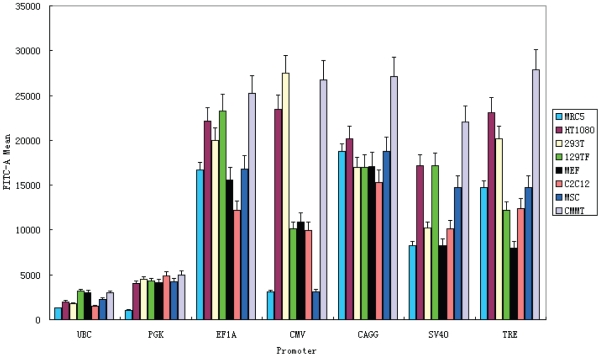
Flow cytometry measurement of GFP fluorescence in eight mammalian cell types transduced with lentiviral vectors carrying GFP reporter driven by promoters of interest. Six mammalian constitutive promoters were tested, along with the doxycycline-inducible TRE promoter at maximal induction.

For the six mammalian constitutive promoters, each one was inserted into a lentiviral expression vector in front of the GFP reporter [11]. After the packaging of each vector into virus, cells were infected by the virus at low titer such that only a small fraction of cells are transduced. This ensures that the majority of transduced cells contain one viral integration per cell. The lentiviral vector also carries puromycin resistance, which was used to select for transduced cells. We then quantitated GFP intensity by flow cytometry (Figure 1). It showed that UBC is consistently the weakest promoter in all the cell types while PGK is also consistently weak, though typically stronger than UBC. By contrast, EF1A and CAGG promoters are consistent strong in all the cell types, with EF1A being slightly stronger in some cell types and slightly weaker in others. SV40 promoter is also fairly strong, though generally somewhat weaker than EF1A and CAGG. While there is cell type to cell type variability for all the promoters, CMV promoter is the most variable, being very strong in some cell types (*e.g.*, 293T and CMMT) and rather weak in others (*e.g.*, MRC5 and MSC). This variability is consistent with the silencing of this promoter in some cells as reported before [12], [13], [14].

**Figure 2:**
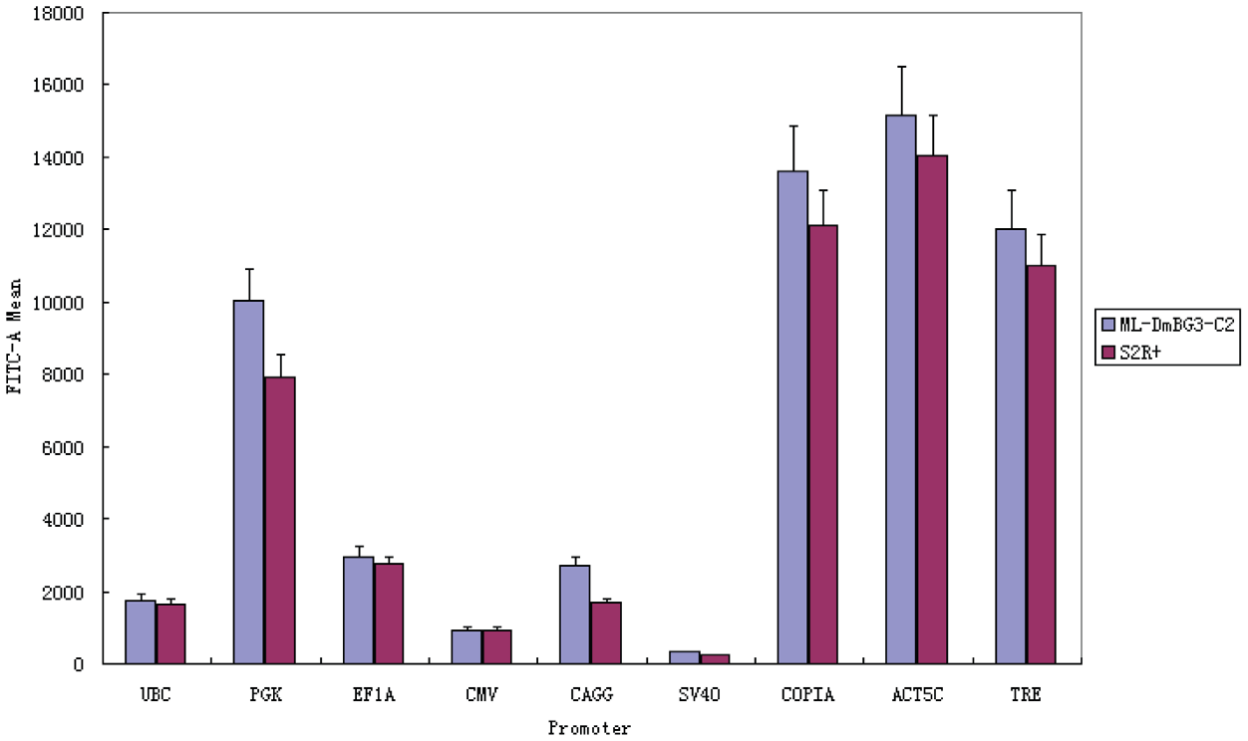
Flow cytometry measurement of GFP fluorescence in two *Drosophila* cell types transduced with lentiviral vectors carrying GFP reporter driven by promoters of interest. Two *Drosophila* constitutive promoters and six mammalian constitutive promoters were tested, along with the doxycycline-inducible TRE promoter at maximal induction.

We then examined COPIA and ACT5C promoters in the two types of *Drosophila* cells. In our hands, lentiviral transduction killed *Drosophila* cells. We therefore used conventional transient transfection to deliver the vectors into the cells. We took care to use identical transfection conditions so as to achieve comparable transfection efficiencies between the COPIA-containing construct and the ACT5C-containing construct. The two promoters produced similar GFP signals in C2 and S2R+ cells, with ACT5C being slightly stronger than COPIA (Figure 2).

To examine whether mammalian promoters could function in fly cells, we also transfected the six mammalian promoters into C2 and S2R+ cells. Surprisingly, with the exception of SV40, all the mammalian promoters produced above-background levels of GFP signals, with PGK producing a level of expression that approaches that of COPIA and ACT5C (Figure 2). It is interesting that PGK, which is a weak promoter in mammalian cells, can behave as a fairly strong promoter in fly cells. It is also interesting that mammalian promoters could be functionally conserved, at least to some extent, in such evolutionary divergent host cells.

One caveat of the flow cytometry measurement of GFP intensity is that the stability of the GFP transcript and/or protein may differ from cell type to cell type. As such, the comparison of GFP intensity across cell types might not be a true reflection of promoter strength in different cell types. However, the comparison of GFP intensity from different promoters within a cell type should reflect promoter strength.

**Figure 3:**
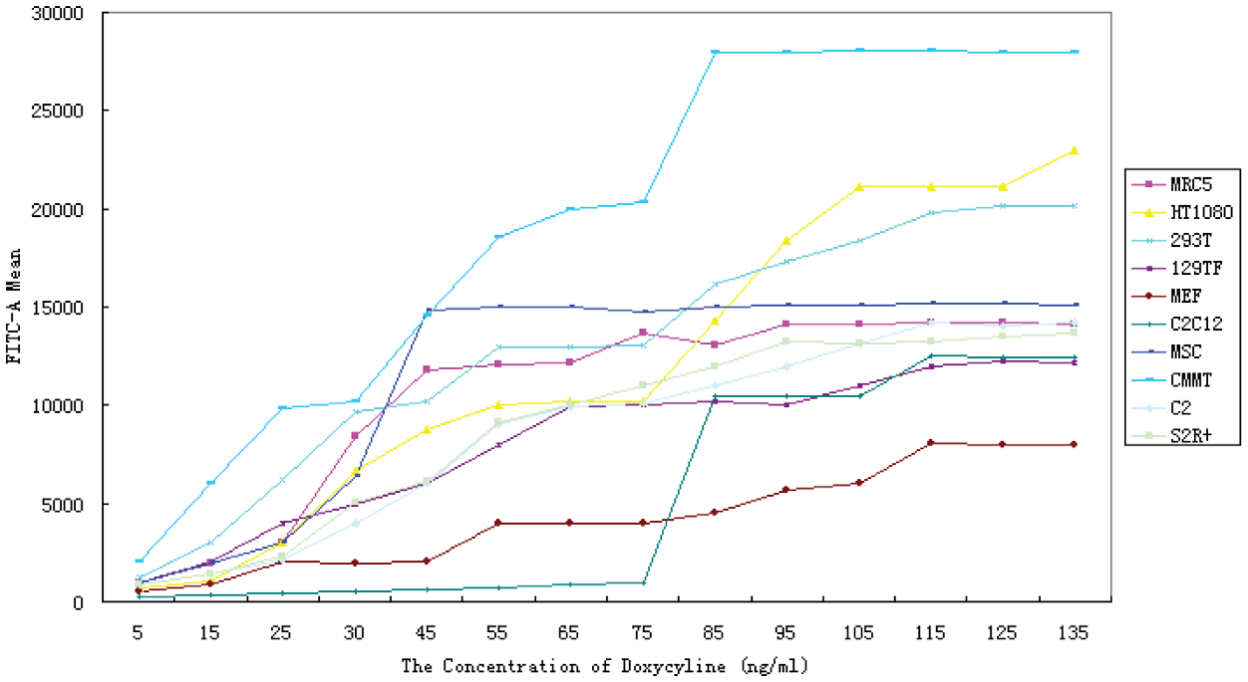
Induction of TRE promoter driving GFP under different doses of doxycycline. Eight mammalian cell types and two *Drosophila* cell types were used in the analysis. GFP fluorescence is quantitated by flow cytometry.

We next examine the behavior of the rtTA-TRE inducible system in the above mammalian and *Drosophila* cell types. In addition to the TRE lentivirus, we also transduced mammalian cells (or transfected *Drosophila* cells) with a vector carrying the rtTA gene driven by the EF1A promoter. This vector also carries neomycin resistance, which was used to select for transduced mammalian cells. A range of doxycycline concentrations were used to induce the TRE promoter, followed by flow cytometry analysis of GFP intensity (Figure 3). At zero concentration of doxycycline, GFP expression is essentially undetectable in all cell types. At nonzero drug concentrations, different cell types responded differently in terms of drug sensitivity and maximum expression level. For example, CMMT cells turned on GFP at much lower doxycycline concentrations as compared to C2C12 cells. GFP expression reached maximum for all cell types at about 110 ng/ml doxycycline concentration, though some cell types (*e.g.*, MSC) reached plateau at a much lower doxycycline concentration. The maximum expression level from the TRE promoter in any of the cell types is comparable to, or only slightly less than, a strong constitutive promoter such as EF1A or CAGG in that cell type (compare maximum TRE activities with that of other promoters in Figures 1 and 2). Thus, the inducible rtTA-TRE system can achieve high levels of gene expression while also affording a means of tight regulation.

## Materials and Methods

Plasmids containing promoters are obtained from the following sources: EF1A, SV40, PGK, CAGG and UBC from published sources [11], [15], [16], CMV from pEGFP-N1 vector of Clontech, ACT5C from pUC-act-DHFR vector of Drosophila Genomics Resources Center, COPIA from p8HCO of Drosophila Genomics Resources Center [17], TRE from pBI-G vector of Clontech. Sequences for these promoters are given in Supporting Information, Text S1. Cells are obtained from the following sources: C2C12, MRC5, MEF, HT1080 and CMMT from ATCC (catalog: CRL-1772, CCL-171, TIB-81, CCL-121 and CRL-6299), 293T from Clontech, MSC from Cyagen Biosciences, ML-DmBG3-c2 and S2R+ from Drosophila Genomics Resources Center (catalog: 62 and 150), and 129TF derived from a 129 strain mouse in our lab.

Promoters are amplified by PCR using primers that contain adaptor sequences to facilitate cloning into lentiviral vectors containing puromycin resistance as previous described [11]. The vectors are packaged into virus following previously described procedures [11], and used to infect mammalian cells, leading to the stable integration of the lentiviral DNA into the host genome. Transient transfection of *Drosophila* cells was carried out according to published protocols [18].

Each batch of lentivirus was first tittered by infecting HT1080 cells with serial dilutions of the virus, followed by extended culture under puromycin selection. Within some range of serial dilutions, the vast majority of cells would be killed because they were not infected, with a small number of infected cells growing up as visible colonies after about 10 days. These colonies were counted and the number was use to calculate viral titer. Once an accurate viral titer was obtained, the virus was used to infect target cells at around 5% infection rate. Infected cells were selected with puromycin two days after infection for about 10 days before flow cytometry analysis, which should kill all the uninfected cells based on drug curves we had done on all the target cell types. To confirm that the great majority of cells only had a single viral integration under the above conditions, we co-infected cells with both GFP-bearing virus and virus bearing the gene for red fluorescence protein (RFP) using the same conditions. We observed that doubly infected cells (which display both GFP and RFP) represented <5% of total infected cells. *Drosophila* cells were cultured for four days after transient transfection before subjected to flow cytometry analysis.

For doxycycline induction of the TRE promoter, mammalian cells are treated with doxycycline right after drgu selection while *Drosophila* cells were treated one day after transient transfection. Gene expression was induced within 12 hours and becomes stable after another day of treatment.

Flow cytometry analysis was carried out on populations (as opposed to clones) of cells. GFP fluorescence was measured on a BD LSR II machine, with all the cells measured under the same machine parameters. Each cell type and promoter combination was done with three independent replicas, and each cytometric measurement was done on 100,000 cells.

## Supporting Information

Text S1 Promoter sequences.
(0.03 MB DOC)
