# Supplementary material for: Systematic Comparison of Constitutive Promoters and the Doxycycline-Inducible Promoter
Source: PLoS One. 2010 May 12;5(5):e10611. doi: 10.1371/journal.pone.0010611 (PMC2868906; doi:10.1371/journal.pone.0010611)
Supplement: Text S1 — Promoter sequences. (0.03 MB DOC) [file pone.0010611.s001.doc]

**Text S1.** Promoter sequences

UBC

GGTGCAGCGGCCTCCGCGCCGGGTTTTGGCGCCTCCCGCGGGCGCCCCCCTCCTCACGGCGAGCGCTGCCACGTCAGACGAAGGGCGCAGGAGCGTTCCTGATCCTTCCGCCCGGACGCTCAGGACAGCGGCCCGCTGCTCATAAGACTCGGCCTTAGAACCCCAGTATCAGCAGAAGGACATTTTAGGACGGGACTTGGGTGACTCTAGGGCACTGGTTTTCTTTCCAGAGAGCGGAACAGGCGAGGAAAAGTAGTCCCTTCTCGGCGATTCTGCGGAGGGATCTCCGTGGGGCGGTGAACGCCGATGATTATATAAGGACGCGCCGGGTGTGGCACAGCTAGTTCCGTCGCAGCCGGGATTTGGGTCGCGGTTCTTGTTTGTGGATCGCTGTGATCGTCACTTGGTGAGTTGCGGGCTGCTGGGCTGGCCGGGGCTTTCGTGGCCGCCGGGCCGCTCGGTGGGACGGAAGCGTGTGGAGAGACCGCCAAGGGCTGTAGTCTGGGTCCGCGAGCAAGGTTGCCCTGAACTGGGGGTTGGGGGGAGCGCACAAAATGGCGGCTGTTCCCGAGTCTTGAATGGAAGACGCTTGTAAGGCGGGCTGTGAGGTCGTTGAAACAAGGTGGGGGGCATGGTGGGCGGCAAGAACCCAAGGTCTTGAGGCCTTCGCTAATGCGGGAAAGCTCTTATTCGGGTGAGATGGGCTGGGGCACCATCTGGGGACCCTGACGTGAAGTTTGTCACTGACTGGAGAACTCGGGTTTGTCGTCTGGTTGCGGGGGCGGCAGTTATGCGGTGCCGTTGGGCAGTGCACCCGTACCTTTGGGAGCGCGCGCCTCGTCGTGTCGTGACGTCACCCGTTCTGTTGGCTTATAATGCAGGGTGGGGCCACCTGCCGGTAGGTGTGCGGTAGGCTTTTCTCCGTCGCAGGACGCAGGGTTCGGGCCTAGGGTAGGCTCTCCTGAATCGACAGGCGCCGGACCTCTGGTGAGGGGAGGGATAAGTGAGGCGTCAGTTTCTTTGGTCGGTTTTATGTACCTATCTTCTTAAGTAGCTGAAGCTCCGGTTTTGAACTATGCGCTCGGGGTTGGCGAGTGTGTTTTGTGAAGTTTTTTAGGCACCTTTTGAAATGTAATCATTTGGGTCAATATGTAATTTTCAGTGTTAGACTAGTAAA

PGK

TTCTACCGGGTAGGGGAGGCGCTTTTCCCAAGGCAGTCTGGAGCATGCGCTTTAGCAGCCCCGCTGGGCACTTGGCGCTACACAAGTGGCCTCTGGCCTCGCACACATTCCACATCCACCGGTAGGCGCCAACCGGCTCCGTTCTTTGGTGGCCCCTTCGCGCCACCTTCTACTCCTCCCCTAGTCAGGAAGTTCCCCCCCGCCCCGCAGCTCGCGTCGTGCAGGACGTGACAAATGGAAGTAGCACGTCTCACTAGTCTCGTGCAGATGGACAGCACCGCTGAGCAATGGAAGCGGGTAGGCCTTTGGGGCAGCGGCCAATAGCAGCTTTGCTCCTTCGCTTTCTGGGCTCAGAGGCTGGGAAGGGGTGGGTCCGGGGGCGGGCTCAGGGGCGGGCTCAGGGGCGGGGCGGGCGCCCGAAGGTCCTCCGGAGGCCCGGCATTCTGCACGCTTCAAAAGCGCACGTCTGCCGCGCTGTTCTCCTCTTCCTCATCTCCGGGCCTTTCGACCT

EF1A

GGCTCCGGTGCCCGTCAGTGGGCAGAGCGCACATCGCCCACAGTCCCCGAGAAGTTGGGGGGAGGGGTCGGCAATTGAACCGGTGCCTAGAGAAGGTGGCGCGGGGTAAACTGGGAAAGTGATGTCGTGTACTGGCTCCGCCTTTTTCCCGAGGGTGGGGGAGAACCGTATATAAGTGCAGTAGTCGCCGTGAACGTTCTTTTTCGCAACGGGTTTGCCGCCAGAACACAGGTAAGTGCCGTGTGTGGTTCCCGCGGGCCTGGCCTCTTTACGGGTTATGGCCCTTGCGTGCCTTGAATTACTTCCACCTGGCTGCAGTACGTGATTCTTGATCCCGAGCTTCGGGTTGGAAGTGGGTGGGAGAGTTCGAGGCCTTGCGCTTAAGGAGCCCCTTCGCCTCGTGCTTGAGTTGAGGCCTGGCCTGGGCGCTGGGGCCGCCGCGTGCGAATCTGGTGGCACCTTCGCGCCTGTCTCGCTGCTTTCGATAAGTCTCTAGCCATTTAAAATTTTTGATGACCTGCTGCGACGCTTTTTTTCTGGCAAGATAGTCTTGTAAATGCGGGCCAAGATCTGCACACTGGTATTTCGGTTTTTGGGGCCGCGGGCGGCGACGGGGCCCGTGCGTCCCAGCGCACATGTTCGGCGAGGCGGGGCCTGCGAGCGCGGCCACCGAGAATCGGACGGGGGTAGTCTCAAGCTGGCCGGCCTGCTCTGGTGCCTGGTCTCGCGCCGCCGTGTATCGCCCCGCCCTGGGCGGCAAGGCTGGCCCGGTCGGCACCAGTTGCGTGAGCGGAAAGATGGCCGCTTCCCGGCCCTGCTGCAGGGAGCTCAAAATGGAGGACGCGGCGCTCGGGAGAGCGGGCGGGTGAGTCACCCACACAAAGGAAAAGGGCCTTTCCGTCCTCAGCCGTCGCTTCATGTGACTCCACGGAGTACCGGGCGCCGTCCAGGCACCTCGATTAGTTCTCGAGCTTTTGGAGTACGTCGTCTTTAGGTTGGGGGGAGGGGTTTTATGCGATGGAGTTTCCCCACACTGAGTGGGTGGAGACTGAAGTTAGGCCAGCTTGGCACTTGATGTAATTCTCCTTGGAATTTGCCCTTTTTGAGTTTGGATCTTGGTTCATTCTCAAGCCTCAGACAGTGGTTCAAAGTTTTTTTCTTCCATTTCAGGTGTCGTGA

CMV

TAGTTATTAATAGTAATCAATTACGGGGTCATTAGTTCATAGCCCATATATGGAGTTCCGCGTTACATAACTTACGGTAAATGGCCCGCCTGGCTGACCGCCCAACGACCCCCGCCCATTGACGTCAATAATGACGTATGTTCCCATAGTAACGCCAATAGGGACTTTCCATTGACGTCAATGGGTGGAGTATTTACGGTAAACTGCCCACTTGGCAGTACATCAAGTGTATCATATGCCAAGTACGCCCCCTATTGACGTCAATGACGGTAAATGGCCCGCCTGGCATTATGCCCAGTACATGACCTTATGGGACTTTCCTACTTGGCAGTACATCTACGTATTAGTCATCGCTATTACCATGGTGATGCGGTTTTGGCAGTACATCAATGGGCGTGGATAGCGGTTTGACTCACGGGGATTTCCAAGTCTCCACCCCATTGACGTCAATGGGAGTTTGTTTTGGCACCAAAATCAACGGGACTTTCCAAAATGTCGTAACAACTCCGCCCCATTGACGCAAATGGGCGGTAGGCGTGTACGGTGGGAGGTCTATATAAGCAGAGCTGGTTTAGTGAACCGTCAGATC

CAGG

ACTAGTTATTAATAGTAATCAATTACGGGGTCATTAGTTCATAGCCCATATATGGAGTTCCGCGTTACATAACTTACGGTAAATGGCCCGCCTGGCTGACCGCCCAACGACCCCCGCCCATTGACGTCAATAATGACGTATGTTCCCATAGTAACGCCAATAGGGACTTTCCATTGACGTCAATGGGTGGAGTATTTACGGTAAACTGCCCACTTGGCAGTACATCAAGTGTATCATATGCCAAGTACGCCCCCTATTGACGTCAATGACGGTAAATGGCCCGCCTGGCATTATGCCCAGTACATGACCTTATGGGACTTTCCTACTTGGCAGTACATCTACGTATTAGTCATCGCTATTACCATGGTCGAGGTGAGCCCCACGTTCTGCTTCACTCTCCCCATCTCCCCCCCCTCCCCACCCCCAATTTTGTATTTATTTATTTTTTAATTATTTTGTGCAGCGATGGGGGCGGGGGGGGGGGGGGGGCGCGCGCCAGGCGGGGCGGGGCGGGGCGAGGGGCGGGGCGGGGCGAGGCGGAGAGGTGCGGCGGCAGCCAATCAGAGCGGCGCGCTCCGAAAGTTTCCTTTTATGGCGAGGCGGCGGCGGCGGCGGCCCTATAAAAAGCGAAGCGCGCGGCGGGCGGGGAGTCGCTGCGACGCTGCCTTCGCCCCGTGCCCCGCTCCGCCGCCGCCTCGCGCCGCCCGCCCCGGCTCTGACTGACCGCGTTACTCCCACAGGTGAGCGGGCGGGACGGCCCTTCTCCTCCGGGCTGTAATTAGCGCTTGGTTTAATGACGGCTTGTTTCTTTTCTGTGGCTGCGTGAAAGCCTTGAGGGGCTCCGGGAGGGCCCTTTGTGCGGGGGGAGCGGCTCGGGGGGTGCGTGCGTGTGTGTGTGCGTGGGGAGCGCCGCGTGCGGCTCCGCGCTGCCCGGCGGCTGTGAGCGCTGCGGGCGCGGCGCGGGGCTTTGTGCGCTCCGCAGTGTGCGCGAGGGGAGCGCGGCCGGGGGCGGTGCCCCGCGGTGCGGGGGGGGCTGCGAGGGGAACAAAGGCTGCGTGCGGGGTGTGTGCGTGGGGGGGTGAGCAGGGGGTGTGGGCGCGTCGGTCGGGCTGCAACCCCCCCTGCACCCCCCTCCCCGAGTTGCTGAGCACGGCCCGGCTTCGGGTGCGGGGCTCCGTACGGGGCGTGGCGCGGGGCTCGCCGTGCCGGGCGGGGGGTGGCGGCAGGTGGGGGTGCCGGGCGGGGCGGGGCCGCCTCGGGCCGGGGAGGGCTCGGGGGAGGGGCGCGGCGGCCCCCGGAGCGCCGGCGGCTGTCGAGGCGCGGCGAGCCGCAGCCATTGCCTTTTATGGTAATCGTGCGAGAGGGCGCAGGGACTTCCTTTGTCCCAAATCTGTGCGGAGCCGAAATCTGGGAGGCGCCGCCGCACCCCCTCTAGCGGGCGCGGGGCGAAGCGGTGCGGCGCCGGCAGGAAGGAAATGGGCGGGGAGGGCCTTCGTGCGTCGCCGCGCCGCCGTCCCCTTCTCCCTCTCCAGCCTCGGGGCTGTCCGCGGGGGGACGGCTGCCTTCGGGGGGGACGGGGCAGGGCGGGGTTCGGCTTCTGGCGTGTGACCGGCGGCTCTAGAGCCTCTGCTAACCATGTTCATGCCTTCTTCTTTTTCCTACAGCTCCTGGGCAACGTGCTGGTTATTGTGCTGTCTCATCATTTTGGCAAAGAATTC

SV40

CTGTGGAATGTGTGTCAGTTAGGGTGTGGAAAGTCCCCAGGCTCCCCAGCAGGCAGAAGTATGCAAAGCATGCATCTCAATTAGTCAGCAACCAGGTGTGGAAAGTCCCCAGGCTCCCCAGCAGGCAGAAGTATGCAAAGCATGCATCTCAATTAGTCAGCAACCATAGTCCCGCCCCTAACTCCGCCCATCCCGCCCCTAACTCCGCCCAGTTCCGCCCATTCTCCGCCCCATGGCTGACTAATTTTTTTTATTTATGCAGAGGCCGAGGCCGCCTCTGCCTCTGAGCTATTCCAGAAGTAGTGAGGAGGCTTTTTTGGAGGCCTAGGCTTTTGCAAAAAGCT

COPIA

ATCTGTTGGAATATACTATTCAACCTACAAAAATAACGTTAAACAACACTACTTTATATTTGATATGAATGGCCACACCTTTTATGCCATAAAACATATTGTAAGAGAATACCACTCTTTTTATTCCTTCTTTCCTTCTTGTACGTTTTTTGCTGTGAGTAGGTCGTGGTGCTGGTGTTGCAGTTGAAATAACTTAAAATATAAATCATAAAACTCAAACATAAACTTGACTATTTATTTATTTATTAAGAAAGGAAATATAAATTATAAATTACAACAGGTT

ACT5C

GGAAGTACACTCTTCATGGCGATATACAAGACACACACAAGCACGAACACCCAGTTGCGGAGGAAATTCTCCGTAAATGAAAACCCAATCGGCGAACAATTCATACCCATATATGGTAAAAGTTTTGAACGCGACTTGAGAGCGGAGAGCATTGCGGCTGATAAGGTTTTAGCGCTAAGCGGGCTTTATAAAACGGGCTGCGGGACCAGTTTTCATATCACTACCGTTTGAGTTCTTGTGCTGTGTGGATACTCCTCCCGACACAAAGCCGCTCCATCAGCCAGCAGTCGTCTAATCCAGAGACACC

TRE

GGTACCGAGCTCGACTTTCACTTTTCTCTATCACTGATAGGGAGTGGTAAACTCGACTTTCACTTTTCTCTATCACTGATAGGGAGTGGTAAACTCGACTTTCACTTTTCTCTATCACTGATAGGGAGTGGTAAACTCGACTTTCACTTTTCTCTATCACTGATAGGGAGTGGTAAACTCGACTTTCACTTTTCTCTATCACTGATAGGGAGTGGTAAACTCGACTTTCACTTTTCTCTATCACTGATAGGGAGTGGTAAACTCGACTTTCACTTTTCTCTATCACTGATAGGGAGTGGTAAACTCGACCTATATAAGCAGAGCTCGTTTAGTGAACCGTCAGATCGCCTGGAGACGCCATCCACGCTGTTTTGACCTCCATAGAAGACACCGGGACCGATCCAGCCTCCGCGGCCCCGAATTG
